# Examining the Potential Synergistic Effects Between Mindfulness Training and Psychedelic-Assisted Therapy

**DOI:** 10.3389/fpsyt.2021.707057

**Published:** 2021-08-11

**Authors:** Maria Eleni Eleftheriou, Emily Thomas

**Affiliations:** Department of Clinical Psychopharmacology, University College London, London, United Kingdom

**Keywords:** psychedelic therapy, mindfulness, meditation, MBSR, ayahuasca, 5-MeO-DMT, psilocybin, decentering

## Abstract

Mindfulness-based interventions and psychedelic-assisted therapy have been experimentally utilised in recent years as alternative treatments for various psychopathologies with moderate to great success. Both have also demonstrated significant post-acute and long-term decreases in clinical symptoms and enhancements in well-being in healthy participants. These two therapeutic interventions share various postulated salutogenic mechanisms, such as the ability to alter present-moment awareness and anti-depressive action, *via* corresponding neuromodulatory effects. Recent preliminary evidence has also demonstrated that psychedelic administration can enhance mindfulness capacities which has already been demonstrated robustly as a result of mindfulness-based interventions. These shared mechanisms between mindfulness-based interventions and psychedelic therapy have led to scientists theorising, and recently demonstrating, synergistic effects when both are used in combination, in the form of potentiated therapeutic benefit. These synergistic results hold great promise but require replication in bigger sample groups and better controlled methodologies, to fully delineate the effect of set and setting, before they can be extended onto clinical populations.

## Introduction

### Mindfulness Meditation and Mindfulness-Based Interventions

Meditation refers to a form of cognitive training involving the regulation of attention by focusing it on a specific goal, which varies depending on the type of meditation. Different types of meditation have arisen from various spiritual traditions, with cognitive neuroscience focusing on techniques deriving from Buddhist traditions and their secularised forms, especially mindfulness meditation (1). Mindfulness meditation is a subtype of meditation, developed in the traditions of Theravada and Tibetan Buddhism (2), which aims specifically at cultivating mindfulness (“sati” in Pali), defined as “paying attention in a particular way: on purpose, in the present moment and non-judgementally” (3). Mindfulness meditation practise includes various subtypes, such as focused attention and open awareness meditation, with the former explicitly focusing attention on a specific object while resisting engagement with emerging thoughts, while the latter aims to cultivate non-selective awareness of internal and external stimuli (4). These practises aim to cultivate meta-awareness, a process by which mental episodes of mind wandering are noted and attention is re-focused back onto the original goal of the meditation (e.g., observing one's breath) (5). Mindfulness meditation has demonstrated benefits in healthy individuals, such as enhanced attentional regulation and positive affect (6, 7), and mindfulness training has also been applied to treat conditions such as addiction, anxiety and pain, with moderate success (8–12).

Mindfulness meditation has been implemented into certain psychotherapeutic models, giving rise to mindfulness-based interventions (MBIs), which combine formal and informal mindfulness techniques, such as sitting meditation and the “body scan,” with elements of cognitive behavioural therapy (CBT), usually over an 8-week period (13). These MBIs have been modified to specifically target certain psychopathologies: for example, mindfulness-based stress reduction [MBSR; (14)] is often utilised to treat anxiety and depression, while mindfulness-based relapse prevention [MBRP; (15)] is applied to prolong abstinence in individuals at risk of substance use disorder. These mindfulness-based interventions have been successfully applied to a plethora of physical and mental conditions and have yielded moderate to large effect sizes for treating anxiety, depression and stress, whereas conditions such as chronic pain incurred little benefit from them (11). However, due to the relative novelty of these interventions within Western science, most of these clinical studies suffer from low methodological quality, and thus benefits may potentially be overstated.

Certain psychological mechanisms have been proposed as subservient of mindfulness-mediated salutogenic effects, such as enhancements in cognitive flexibility, decentring and emotional regulation, as well as neuroplastic changes (16–20). Short-term and long-term neurological changes have been observed in cross-sectional and longitudinal studies, comparing meditators to meditation-naïve controls, which demonstrated changes in connectivity, baseline activation patterns as well as white or grey matter density in various regions (18). Recurrently implicated regions involve the thalamus, a crucial brain region subserving sensory gating, and various nodes of the default-mode network (DMN), a wide-spread network thought to subserve self-related cognition (21). However, to date, these neurological changes have only been tentatively linked to behavioural changes and have not been causally implicated in meditation's benefits on well-being and psychopathology (22, 23).

### The Psychedelic Renaissance

The term “psychedelics” refers to wide class of psychoactive compounds, most of them naturally-occurring, capable of producing alterations in consciousness *via* their pharmacological effects on diverse neurotransmitter systems. “Classical” serotonergic psychedelics, including psilocybin and LSD, act as agonists on the serotonin system, with relative selectivity for the 5HT_2A_ receptor (24), whilst dissociative psychedelics, such as ketamine, hijack the glutamatergic system, acting as NMDAR antagonists (25). Consumption of such compounds leads to altered states of consciousness, characterised by sensory perturbations, including pseudo-hallucinations, as well as the re-emergence of emotional content, which can lead to long-lasting insights after the experience has ended (26–28).

Psychedelics have recently been investigated as an alternative treatment for treating various psychiatric conditions with promising results. Most of these studies however suffer from low methodological validity and are mostly open-label, yet they have yielded preliminary evidence for the capacity of psychedelic substances for treating addiction, depression, anxiety, and eating disorders (29–35). In recent years, several randomised controlled trials (RCTs) have robustly demonstrated ayahuasca, ketamine and psilocybin's potent anti-depressive effects (36–38). Psychedelics have also been shown to repeatedly produce enhancements in well-being in healthy individuals, giving rise to effects such as enhanced mood, altruistic behaviour and prosociality, as well as significant decreases in clinical symptomatology (39–41). Thus, psychedelic-assisted therapy is emerging as a very promising alternative treatment and preventative strategy for numerous psychopathologies, but still requires further corroboration.

The mechanisms underlying the acute and long-lasting effects on psychedelics on well-being are still under debate. Various psychological, behavioural and neuromodulatory mechanisms have been suggested to underlie the observed enhancements in well-being and psychopathology (42, 43). Namely, psychedelics seem to induce a period of “afterglow,” characterised by a sustained positive mood, novel insights and beneficial changes in behaviour after acute effects have subsided (44). FMRI studies have also demonstrated acute and long-lasting neurological effects after psychedelic-administration, including neuromodulatory effects on the DMN, as well as changes in global brain connectivity (45–48). Of these proposed mechanisms, the acute disruption of the DMN, as well as mystical-type experiences, have been strongly correlated with observed benefits, but the remainder still require conclusively testing (49, 50).

### Examining Synergy Between Psychedelics and Mindfulness Training

Mindfulness-based approaches and psychedelics share various phenomenological and neuropsychological effects, and it has been proposed that a combined approach may yield greater enhancement in clinical outcomes and well-being than each intervention applied alone (51, 52). Both mindfulness meditation and psychedelics have the potential of inducing altered states of consciousness, which may be lacking similar elements of self-awareness, such as narrative and multisensory aspects of self-consciousness (1). Intense “peak” experiences, characterised by feelings of cosmic unity and powerful insights, can occur as a result of psychedelic-administration, in what is termed drug-induced ego dissolution (DIED) (53), and occasionally in experienced meditators (54). The occurrence of such mystical-type experiences has been linked to enhancements in well-being for both interventions (50, 55).

Recent studies have also demonstrated the capacity of various psychedelics to enhance trait mindfulness post-acutely, even in the absence of a theoretical or practical context of mindfulness training, evidencing a complementary relationship between mindfulness-training and psychedelics (40, 52, 56–59). Other shared mechanisms, including anti-depressive, anxiolytic and neuromodulatory effects, suggest that a combined approach of psychedelic-assisted mindfulness-based interventions could yield synergistic effects (51). This was effectively demonstrated in three recent studies, which, utilising a combined design, produced beneficial trait-alterations, as well as enhancements in mood, satisfaction with life and behavioural change, which were more significant and longer-lasting than previously observed changes, when each intervention was used in isolation (60–62).

In the following sections I summarise the neural and psychological therapeutic mechanisms shared between mindfulness-based interventions and psychedelic-assisted treatment and review evidence demonstrating synergistic effects between the two. These shared salutogenic mechanisms might underlie synergistic benefits observed in three recent studies which combined psychedelic administration with mindfulness training, reviewed in detail in section Three Recent Studies Utilising a Combined Methodology. Additionally, I review recent preliminary evidence indicating psychedelics' trait mindfulness-enhancing potential and, finally, address several limitations and future considerations for the field.

## Shared Salutogenic Mechanisms Between Mindfulness Training and Psychedelics

Various mutually non-exclusive mechanisms have been theorised to subserve the salutogenic effects of both mindfulness-based interventions and psychedelic-assisted therapy. These include psychological, behavioural and neuromodulatory mechanisms, such as emotional regulation, anti-depression and acute disruption of the DMN. The complementary activation by psychedelic administration and mindfulness interventions of these shared beneficial mechanisms could potentiate the therapeutic benefit of either intervention alone in terms of both intensity and duration.

### Anti-depressive and Anxiolytic Effects

Mindfulness-based cognitive therapy (MBCT), as well as psychedelic-assisted therapy, have both been successfully applied to treat depression with moderate to large effect sizes. Randomised clinical trials (RCTs) of psilocybin-assisted therapy demonstrated a significant reduction of depressive symptoms in terminally ill cancer patients, as well as in treatment-resistant depression (29, 30, 38). Remarkably, these benefits were incurred even after only a single psychedelic administration and were maintained for as long as 6 months. Similarly, ayahuasca's anti-depressive potential has been demonstrated in pilot studies and replicated in a RCT on patients with treatment-resistant depression (33, 36, 63). Ketamine has also demonstrated remarkable and rapid anti-depressant effects, manifesting as early as a day after administration, in contrast to the “response lag” observed with traditional antidepressants (37, 42, 64). MBCT, on the other hand, was able to significantly reduce depression and anxiety symptoms, to sub-clinical levels, and produced a large therapeutic effect size for all three conditions of anxiety, depression and stress (11).

Both interventions have also successfully demonstrated reductions in clinical symptomatology in healthy individuals (65, 66). Uthaug et al. recorded significant reductions in anxiety, depression and stress scores, following naturalistic administration of ayahuasca and 5-MeO-DMT (40, 58). Mindfulness training has also been linked to enhanced well-being and lower psychopathology, in both longitudinal and cross-sectional studies (1, 18). These anti-depressive and anxiolytic effects are thought to in turn be mediated by enhancements in decentring and cognitive flexibility, in the case of mindfulness intervention (16, 19), vs. mystical experiences and disrupted cortical connectivity in the case of psychedelic-assisted therapy (49, 50).

Neurogenesis may also contribute to these effects, as psilocybin, ketamine as well as β-carbolines present in the ayahuasca brew, have all been shown to enhance neurogenesis in animal studies (42, 67, 68). Neurogenesis has been shown to be impaired in depression, and critically underlies the effect of antidepressant medication (69). Additionally, psychedelics may provide relief in depression and anxiety by enhancing engagement with therapy, which can be remarkably low in mentally ill patients, particularly in the case of treatment-resistant depression (42, 43, 70). Thus, enhanced and longer-lasting anti-depressive and anxiolytic effects may be yielded by a combined approach, due to a complementary enhancement of the same salutogenic mechanisms by mindfulness- and psychedelic-based interventions (51), or due to a psychedelic-induced enhancement in MBCT engagement.

### Mystical Experiences

Mystical-type experiences, also referred to as “peak,” visionary and quantum change experiences, refer to sudden intensely meaningful experiences, often involving an alteration in self-awareness and feelings of unity and transcendence of time and space (71–73). These occurrences are usually extremely rare and profoundly personally meaningful, and often lead to long-lasting transformations in psychology and behaviour (28, 74, 75). Mystical experiences were originally reported within a ritualistic context, and were a result of fasting, sleep deprivation, dancing and chanting (76), but they can also occur in experienced mediators (54), and more reliably as a result of psychedelic-administration, in a phenomenon termed “Drug-Induced Ego Dissolution” (53, 77).

Phenomenologically, the qualitative experience of psychedelic- vs. meditation-induced mystical experiences can be quite different, with psychedelic-induced experiences being often characterised by rich sensory content, which is usually sparse in meditative states. However, both states have been shown to give rise to disruptions in narrative and multisensory aspects of self-consciousness, with meditators albeit inducing those voluntarily (1). Whereas, psilocybin has been shown to reliably yield a mystical-type experience, at a rate of up to 60%, in a dose-dependent manner (78, 79), mindfulness meditation can only rarely give rise to self-transcendence in very experienced meditators (80, 81). Nevertheless, in an extensive survey of Western Buddhists, more than 75% of long-term meditators reported having experienced profound changes in self-perception at some point in their lives, as a result of their meditation practise (82).

The intensity of mystical experiences has been strongly linked with the magnitude of psychedelic-interventions' therapeutic effect in numerous studies, where it was shown to be predictive of positive outcomes for depression, addiction and well-being (28, 36, 49, 50, 74, 83, 84), and has been proposed as the treatment-mediating mechanism in various models of psychedelic-assisted therapy (26, 44). Valuable insights gleaned during the acute experience seem to have a transformational effect after the acute effects have subsided, as was demonstrated for addiction and depression, with nicotine addicts reporting seeing themselves as “smoking gargoyles,” and realising the harm they had been inflicting on their families, and depressed patients facing overwhelming and difficult memories and emotions that had been previously “hidden” in their subconscious (41, 85). The benefits elicited by mindfulness-based interventions have also been linked to acute mystical-type experiences arising during the meditative state (55). However, the long-term enhancements in well-being demonstrated by these approaches are thought to be due to incremental improvements in attentional control and emotion regulation, giving rise to a gradual shift in self-perception-a phenomenon termed “decentring,” explained in the next section (18).

In recent studies, reviewed below (section Psychedelics: A “Gateway” Into Meditative Practises?), the intensity of the acute psychedelic experience was shown to correlate with enhancements in mindfulness capacities (40, 58). Additionally, the administration of psilocybin in the context of mindfulness training led to a significantly stronger acute mystical experience (60, 61). Rates for a “complete” mystical experience (corresponding to >60% in the Mysticism Scale) were remarkably high in both Griffiths' and Smigielski's studies for the experimental groups and were significantly higher than previously reported rates of “complete” mystical experiences due to psilocybin, outside of a mindfulness context (86, 87). Hierarchical regression analyses demonstrated that the intensity of mystical experience predicted most positive shifts in trait measures, observed long-term in the aforementioned studies. Thus, intensified mystical experiences provides a good candidate mechanism for how mindfulness training, and high spiritual support, can enhance the beneficial effects of psychedelic therapy.

### Psychological Mechanisms

Various psychological mechanisms have been implicated in the beneficial effects of mindfulness meditation and related interventions, as well as psychedelic-assisted therapy, such as enhancements in mood, emotion regulation and decentring ability. Mindfulness meditation and mindfulness-based interventions can augment positive affect in healthy and clinical populations acutely and long-term (10, 88) and a similar mood-enhancing potential has been demonstrated for psychedelics such as psilocybin (29, 38, 60, 83, 87). Additionally, increased life satisfaction and sense of purpose has been demonstrated long-term after both interventions and was particularly profound in combined designs (60, 61).

Enhancements in prosociality are thought to contribute as well, which have been reported to result after both psychedelic-assisted and mindfulness-based interventions (28, 89). Individuals were shown to foster kindness, empathy and compassion after mindfulness training and psilocybin administration (90–92), while the latter also led to higher scores in facets of interpersonal closeness and trait forgiveness (60). Furthermore, positive changes in behaviour are also thought to contribute to both prosociality and mood, and enhancements in altruistic and value-based behaviour have been reported as a result of both interventions (41, 60, 93). Behavioural activation was shown to be strongly associated with ayahuasca's anti-depressant effects and may help extend acute benefits into long-term enhancements in well-being (94). In the case of mindfulness training, enhanced well-being is thought to result from a gradual shift in self-perception, entailing a less rigid and omnipresent self-concept, (51, 95), whilst psychedelic-mediated benefit is thought to be due to the acute psychedelic experiences, often involving feelings of bliss and cosmic unity, and the emotional content and insights that emerge from it (26, 49).

Decentring capacity has been crucially linked to psychopathology in various psychiatric disorders and shown to mediate treatment effects of mindfulness-based interventions (96). Decentring refers to the ability to assume a detached, objective stance towards present-moment awareness, whereby emerging thoughts and emotions are considered temporary events of the mind (97). Pathological rumination and reduced decentring ability have been linked with many psychiatric disorders, including major depression, eating and cocaine use disorders (98), while both psychological and pharmacological treatments, including CBT, antidepressants and mindfulness-based interventions, have all been linked to enhancements in decentring ability (17, 97, 99). Magnified decentring ability was also shown post-acutely after ayahuasca administration (56, 57), suggesting that this may be a shared beneficial effect between psychedelic- and mindfulness-based treatments. Furthermore, the Buddhist concept of non-attachment is similar to decentring and was increased as a result of psilocybin administered within a spiritually supportive context (60, 100). Thus, mindfulness-based interventions and psychedelic administration may work complementarily to enhance decentring ability and reduce psychopathology.

In the case of mindfulness meditation training, detachment from intrusive thoughts and emotions has been shown to be mediated by heightened emotional and attentional control, at least in the case of novice meditators (18). As meditation is a form of attentional training, it leads to incremental enhancements of executive regulation of cognitive function. Evidence for increased executive control after psychedelic-administration also exists, particularly in the case of ayahuasca (57, 101). However, in the case of psychedelics, enhanced decentring is thought to result from a rapid perturbation of descending executive control over sensory processing, resulting from psychedelics' potential to enhance global brain entropy and disrupt predictive coding (102). Thus, it is theorised that habitual associations and predictions about the environment, such as negative bias observed in major depression, are disrupted, giving rise to a “freer, lighter” mental state, which characterises the post-acute “afterglow” period after psychedelic administration (44, 103).

Recently, “psychological flexibility,” defined as the ability to fully experience the present moment and behave adaptively in a value-based manner, was proposed as the treatment mediator of psychedelic-assisted interventions (75). Psychological and emotional flexibility, and decreased identification with a static sense of self, are explicit aims of Buddhist traditions, such as mindfulness meditation and its secularised forms (1), and enhanced cognitive flexibility has been observed in more advanced mindfulness meditators (18, 104). A long-term mindfulness meditation practise is thought to cultivate an accepting, non-judgemental present-moment awareness (18). These key aspects of acceptance towards present-moment awareness have been crucially linked with psychiatric disorders (105), and acceptance measures were shown to be better predictors of lower psychopathology than even emotional regulation strategies (106). Acceptance facets of mindfulness, however, can be particularly resistant to mindfulness-training and were unchanged even after a 1-month mindfulness meditation retreat (107). Certain psychedelics have been shown to acutely and post-acutely enhance non-judgemental and non-reactive awareness, even outside of a theoretical mindfulness context, and to increase self-compassion long-term (40, 52, 56–59). Psychedelics and mindfulness meditation may thus be able to work synergistically, with psychedelics facilitating rapid enhancements in self-acceptance, which can then be extended in time through a rigorous long-term mindfulness meditation practise.

### Neuromodulatory Effects

Recent fMRI studies have demonstrated transient and long-term neuromodulatory effects as a result of mindfulness meditation practise as well as psychedelic administration. Despite dissociable patterns of neurometabolic changes observed during different types of mindfulness meditation, various nodes of the Default Mode Network (DMN) have been consistently implicated, such as the posterior cingulate cortex (PCC), which showed transient deactivation (18, 108). The DMN is a collection of distant brain regions which get differentially activated during self-related cognition, such as autobiographical memory recollection and mental time-travel (109), and its activity has been shown to correlate with self-reported mind-wandering in the absence of a cognitive task (110). Experienced meditators demonstrated a relative deactivation of the medial prefrontal cortex (mPFC) and PCC when engaged in mindfulness meditation, compared to their brain activity during resting-state (108, 111–113).

Similarly, the administration of various psychedelic drugs has led to transient deactivation of certain DMN hubs, including the mPFC, PCC and anterior cingulate cortex (ACC) (45, 114). This acute disruption of DMN functioning has been hypothesised to act as treatment mediator of psychedelic-assisted therapies (49). Certain psychedelic studies have further correlated these transient connectivity changes within the DMN with subjective measures of drug-induced ego-dissolution (46, 115). This suggests that acute disruptions between nodes of the DMN may be the neural signature underlying mystical-type experiences discussed above (1).

Cross-sectional studies have further implicated the DMN, showing lower baseline activity in the medial prefrontal cortex (mPFC) and PCC in long-term mindfulness meditators compared to meditation-naïve controls (108, 116). In the field of psychedelic neuroscience, reduced mPFC-PCC connectivity as well as DMN deactivation and disintegration have been demonstrated post-acutely after the administration of psilocybin, LSD and ayahuasca (45, 46, 57), while long-term ayahuasca users demonstrated thinning of the PCC (117). These post-acute and long-term reductions in DMN functioning have been theorised to underlie reported long-term changes in self-related cognition (82) through a “detachment from identification with a static sense of self” (118). Decreased rumination might mediate the aforementioned therapeutic effects, as excessive rumination and DMN hyperactivity have been crucially linked in depression (119).

On the contrary, other studies have demonstrated enhanced DMN activity as a result of mindfulness meditation training and psychedelic administration. For example, enhanced activation of the ACC and other prefrontal regions was observed in novice and experienced meditators (120–122), and as a result of ayahuasca administration (33, 63). These neurophenomenological changes may underlie enhancements in attentional and emotional control (18), corroborated by enhanced functional connectivity between the ACC and limbic regions following ayahuasca administration (57). Cortical connexions between executive and limbic areas can become disrupted in clinically depressed individuals (123), and so such enhancements in connectivity may re-instantiate executive control over intrusive emotional thoughts and mediate the anti-depressive effects summarised above.

Furthermore, when psilocybin was administered to long-term mindfulness meditators, it led to enhanced context-dependent DMN flexibility, as the meditators who had received psilocybin were able to downregulate DMN activity significantly more than the comparison group, during mindfulness meditation (101). This suggests an enhancement in the dynamic repertoire of DMN function, which is in line with a plethora of evidence pointing to increases in global brain plasticity and connectivity states as a result of psychedelic administration (47, 48, 124). These increases in dynamic global brain connectivity are thought to underlie observed enhancements in cognitive and psychological flexibility discussed above.

Notably, psilocybin administration led to transiently enhanced functional connectivity between the DMN and the task-positive network (TPN) (45), which is engaged during attention-demanding cognitive tasks and whose activity normally negatively correlates with that of the DMN (109). Interestingly, reduced anti-correlation between these antithetical networks has also been observed as a result of mindfulness meditation training (108, 125) and has been identified as the neural signature of Non-Dual Awareness, which is a type of mindfulness meditation similar to Dzogchen and Mahāmudrā Buddhism, aiming at dissolving the illusory subject-object dichotomy characterising ordinary consciousness (126). Recently Sampedro et al. demonstrated post-acute enhancements in functional connectivity between the PCC of the DMN and the superior rostral ACC of the TPN 24 h after the administration of ayahuasca, and such enhancements were shown to predict improvements in mindfulness capacities when an exploratory correlation analysis was performed (57). Thus, increased long-ranging cortical connexions and harmony between mutually exclusive brain networks may underlie the beneficial effects elicited by mindfulness meditation and psychedelic-assisted therapy, in isolation as well as in combination (102).

## Evidence for Synergistic Effects

### Three Recent Studies Utilising a Combined Methodology

Griffiths et al. were the first to deliver a psychedelic within a context of mindfulness training, administering psilocybin within a program of spiritual training (60). Two psilocybin sessions were combined with different levels of support for spiritual practises, which aimed to cultivate mindfulness through the practise of mindfulness meditation, daily awareness practise and journaling, while instilling spiritual values into everyday experience. Participants were matched for baseline characteristics, including lifetime psychedelic use and frequency of meditation, and randomised into 3 groups of 25 participants each, receiving: (1) a very low dose of psilocybin (1 mg/70 kg-active placebo) coupled with moderate levels of spiritual support, (2) a high dose of psilocybin (20 and 30 mg/70 kg) and moderate spiritual support, or (3) a high dose of psilocybin and high spiritual support. The high spiritual support group received twice the amount of contact hours of guide-participant meetings during the preparatory phase, and nearly five times as many total contact hours, as well as exclusive access to group discussion after the psilocybin sessions, where participants could share their successes and challenges with regular spiritual practises. Psilocybin was administered at 1 and 2 months after the start of spiritual training, in ascending dose, in a relaxed setting, with two guides present at all times who provided non-directive support.

Compared to the active placebo group, both groups which received a high dose of psilocybin reported significantly higher enhancements in mood, well-being and life satisfaction, as assessed by the Persisting Effects Questionnaire (87). Furthermore, only the high dose groups demonstrated positive changes in various trait measures 4 months after the last psilocybin session, such as life meaning, trait forgiveness, daily spiritual experience, interpersonal closeness, gratitude and observer-rated religious sentiments, suggesting a positive shift in attitudes about life and about one self, as well as increased spirituality and altruistic and pro-social behaviour. Some of these trait effects have been demonstrated as a result of long-term formal meditation practise (127–129) and have been linked to increased well-being and lower psychopathology (130–132). Importantly, previous studies utilising psychedelic-administration outside of a mindfulness-enhancing context have failed to show such positive trait changes (83, 87, 133), with the exception of trait openness (74, 134). Thus, this study provides preliminary evidence that psilocybin administration coupled with mindfulness training can enhance each other's beneficial effects on well-being, and produce, potentially salutogenic, positive trait changes.

Similar effects were reported by Smigielski et al., in a study where psilocybin was administered to long-term mindfulness meditators within a Zen meditation retreat context (61). For this study, 39 mindfulness meditators with an average of 5,000 h of mediation experience and 30 meditation retreats were recruited. Half the participants (*n* = 20) received psilocybin (315 μg/kg), while the other half (*n* = 19) received identical-looking lactose capsules. Psilocybin was administered on the fourth day of a highly structured, 5-day Zen meditation retreat setting, during a block of sitting meditation, where participants meditated sitting upright facing a wall with their eyes half open. Four months after the retreat, participants in the experimental group reported significantly higher scores in trait measures denoting appreciation for life, self-acceptance and quest for meaning/sense of purpose, and significantly lower fear of death, as captured by the Life Changes Inventory, Revised [LCI-R; (135)] and confirmed by third person reports. Thus, we see that, in this design, psilocybin administration led to a stronger positive shift in attitudes and behaviour than was produced by the meditation retreat alone.

Crucially, the study designs used makes it impossible to delineate the effect of mindfulness training from that of set and setting on the acute psychedelic experience. Differences in set and setting, which involve factors such as opportunity for preparation, guidance and integration by a trained guide, have been extensively shown to greatly influence the acute psychedelic experience (136, 137). Thus, the safe and supportive setting, as well as the lack of distractions, which characterised the aforementioned studies may have accounted for the high rates of positively experienced ego-dissolution and the low anxiety rates (See Table 1 for details of set and setting).

**Table 1:** Differences in participant characteristics, set and setting.

**Study**	**Substance**	**Dose**	**Age**	**Sample population**	**Prior experience**	**Intentions**	**Setting**
Soler et al. (56)	Ayahuasca	28.82–69.81 mg DMT	43.6 ± 12	Healthy, highly educated	92% Aya-huasca (1–500 times)	• Self-knowledge • Introspection	• Non-religious; dimly-lit room with pre-recorded music • Naturalistic study; experimenters present before, during and after session
Sampedro et al. (57)	Ayahuasca	0.64 mg DMT/kg	38.9 ± 7.8	Healthy, highly educated	100% (62 ± 99 times)	• Personal growth • Introspection	• Non-religious; dimly-lit room; silence alternated with pre-recorded music • Naturalistic study; experimenters present before, during and after session
Soler et al. (52)	Ayahuasca	N.R.	50 ± 14.7	Healthy	100% Ayahuasca	• Self-knowledge • Introspection	• Non-religious; dimly-lit room with pre-recorded music • Naturalistic study; experimenters present before, during and after session
Uthaug et al. (58)	Ayahuasca	189.4–915.4 mg DMT	N.R.	60% Healthy12% Depression1% Anxiety0.04% Personality Disorder0.02% Addiction	57% Ayahuasca; 70.4% Psy	• Self-understanding • Solving issues • Curiosity	• Non-religious; traditional S. American music played; at night • Led by experienced shamans who provided support if needed + ≥2 ayahuasca facilitators • Dutch sample: tipi/big hotel room • Colombian sample: rainforest location/dimly-lit maloca (ceremonial building)
Uthaug et al. (40)	5-MeO-DMT (from *Bufo alvarius*)	20–120 mg	38 ± 5.2	76.2% Healthy9.4% Anxiety7.1% Addiction2.4% Depression2.4% Personality disorder	80% 5-MeO-DMT; 92.9% Psy	• Self- understanding • Solving issues • Healing/trans-formation • Spiritual growth • Find one's purpose	• Non-religious; traditional S. American music played • Sessions led by experienced facilitators and assistants who provided help with integration if needed • Dutch + Czech sample: garden/secluded natural location • Spanish sample: in tipi/rented house
Uthaug et al. (59)	5-MeO-DMT (synthetic)	15–61 mg	33 ± 8.6	Healthy, highly educated	18% 5-MeO-DMT; 100% Psy	• Self-understanding • Curiosity • Solving problems • Explore possibilities	• Individualised 1-to-1 sessions at yoga studio • Held by trained facilitator
Griffiths et al. (60)	Psilocybin	20 + 30 mg/70 kg	42 ± 10.6	Healthy, highly educated	25% Psy	• Exploring spiritual life • Curiosity	• Lying on couch in aesthetic living-room-like envi-ronment; wearing eye mask and headphones playing classical/world music • Supportive but nondirective guides • 1-month preparation and spiritual training with guide; opportunity for integration (and group discussion sessions in the high support group)
Smigielski et al. (61)	Psilocybin	315 μg/kg	52.8 ± 2.5	Healthy meditators, highly educated	45% Psy	N.R.	• Within silent Zen meditation retreat, during block of sitting meditation; musical elements and relaxation periods during acute effects

*Summary of different participant characteristics and conditions used in the studies reviewed in the main text. For controlled studies, participant characteristics and conditions are listed for the experimental group, where a (active dose of a) psychedelic was administered. Prior experience is listed in terms of percentage of participants who had previous experience with the psychedelic used in the respective study, and also includes percentage of participants with prior experience of other psychedelics (Psy), where the first number is not 100%. (N.R., Not reported)*.

Synergistic effects as a result of a combined approach were extended by Dakwar et al. and demonstrated on a clinical population, where the administration of a non-classical psychedelic was combined with a mindfulness-based approach on 55 individuals at risk of cocaine addiction (62). Ketamine was combined with Mindfulness-Based Relapse Prevention (MBRP) in a randomised double-blind placebo controlled clinical trial. MBRP utilises CBT strategies and helps cultivate mindfulness, *via* breathing and visualisation exercises, which is then targeted to resisting the craving effect of drug-related cues. Participants were enrolled onto a 5-week MBRP program, which involved four sessions per day on the first 5 days, followed by one session per week for 4 weeks. On the second day participants in the ketamine group received 40 min of intravenous ketamine infusion (0.5 mg/kg), while the control group received midazolam.

Participants in the ketamine group demonstrated higher post-acute and long-term abstinence rates, in comparison to the control group. 48.2% abstinence was observed for participants in the ketamine group at the end of the study, compared to 10.7% for the control group, and such high rates of abstinence were maintained at 6 months for the experimental group, whilst all participants in the control group relapsed, according to self-reports. This study demonstrated that embedding ketamine administration into a mindfulness-enhancing behavioural training framework successfully extended ketamine's anti-addictive properties, which had previously been reported only transiently, subsiding over several days (138, 139). Thus, Dakwar's study on cocaine users at risk of addiction provides further preliminary evidence suggesting that the combination of mindfulness training and psychedelic administration may work synergistically to amplify the proven salutogenic effects of mindfulness and psychedelics on well-being and mental health. However, specific effects of the mindfulness component to the program cannot be assessed, as there was no behavioural training control condition.

### Psychedelics: A “Gateway” Into Meditative Practises?

In the aforementioned studies, the administration of psilocybin within a context of mindfulness-training significantly enhanced engagement with formal spiritual practises, mindfulness meditation depth and trait mindfulness (60, 61). Spontaneous enhancements in trait mindfulness have also been reported following the administration of various psychedelics, even outside of a mindfulness-enhancing context (40, 52, 56–59). These results (summarised in Table 2) suggest that psychedelics could be utilised as a useful adjunct to aid mindfulness meditation training in novices, but also to deepen the meditation practise of long-term practitioners (150). However, these results remain preliminary and inconclusive, as most studies suffer from very small sample sizes and lack a control group.

**Table 2:** Summary of studies demonstrating enhanced mindfulness after psychedelic administration.

**Study**	**Substance**	**Sample (size)**	**Study design**	**Construct (measure)**	**Findings**
Soler et al. (56)	Ayahuasca	Healthy sample (*n* = 25)	Naturalistic study; Open-label, uncontrolled, WS	Trait mindfulness (FFMQ), Decentering (EQ) MINDSENS composite inde-measured 24 h after	• ↓Judgemental processing of experiences, ↓inner reactivity, ↑Decentring ability, and ↑MINDSENS composite index
Sampedro et al. (57)	Ayahuasca	Healthy sample (*n* = 16)	Naturalist study; Open-label, uncontrolled, WS	Trait mindfulness (FFMQ), Decentering (EQ), MINDSENS composite index, Self-Compassion Questionnaire-measured at 24 h and 2 mo	• ↓Judgemental processing of experiences, ↓inner reactivity, ↑Decentring ability, ↑MINDSENS composite index, and ↑Self-Compassion 24 h after administration
Soler et al. (52)	Ayahuasca	Healthy sample (*n* = 20)	4 sessions (1 week apart) vs. 8-week MBSR programme; WS + BS	Trait mindfulness (FFMQ), Decentering (EQ), MINDSENS composite index-measured 24 h after final ayahuasca session/MBSR programme	• ↓Judgemental processing of experiences • MBSR ↑all 5 mindfulness facets (FFMQ), decentring ability and the MINDSENS composite index
Uthaug et al. (58)	Ayahuasca	Predominantly healthy sample (*n* = 57)	Naturalistic study; Open-label, uncontrolled, WS	Trait mindfulness (FFMQ)- measured at 24 h and 4 weeks	• ↓Judgemental processing of experiences, ↑awareness of, and capacity to observe, present-moment 24 h after administration
Uthaug et al. (40)	5-MeO-DMT (from *Bufo alvarius*)	Predominantly healthy sample (*n* = 75)	Naturalistic study; Open-label, uncontrolled, WS	Trait mindfulness (FFMQ)- measured at 24 h and 4 weeks	• ↓Judgemental processing of experiences and ↑present-moment awareness 4 weeks after administration
Uthaug et al. (59)	5-MeO-DMT (synthetic)	Predominantly healthy sample (*n* = 11)	Naturalistic study; Open-label, uncontrolled, WS	Trait mindfulness (FFMQ)- measured at 24 h and 1 week	• ↓Judgemental processing of experiences 24 h after, which was maintained at 1 week
Griffiths et al. (60)	Psilocybin	Healthy sample (*n* = 75)	2 sessions (or active control) combined with moderate/high intensity 6-month programme of spiri-tual practises; DB, R, PC, WS + BS	Engagement with spiritual practises; Persisting effects on attitudes and behaviour [NAS, ASPIRES, LOT-R, Trait Forgiveness Scale, IOS, NEO PI-R, Hood Mysticism Scale (Lifetime), Death Transcendence Scale, Daily Spiritual Experience Scale, TRIM-18 etc.]	• ↑Non-attachment, ↑Spirituality, ↑Daily trans-cendental experience, ↑Positive Attitudes about Oneself, ↑Engagement in Daily Meditation, ↑Meditation Duration, ↑Frequency of Spiritual Awareness Practise, ↑Engagement in Daily Spiritual Practise and ↑Frequency of Journal Writing 4 months after last psilocybin session in experimental group
Smigielski et al. (61)	Psilocybin	Healthy sample of experienced mind-fulness meditators (*n* = 25)	Single session during 5-day Zen meditation retreat; DB, R, PC, WS + BS	Trait mindfulness (FMI), State mindfulness (TMS), Meditation depth (MEDEQ) and Persisting effects (LCI-R)	• ↑Meditation Depth on the day of administration • ↑Trait Mindfulness and ↑Self-Acceptance 4 months after retreat > control group

*Summary of all studies to date reporting enhancements in mindfulness capacities after the administration of a psychedelic; only relevant findings of these studies are presented (↑denotes increase; ↓indicates decrease in measure). [ASPIRES, Assessment of Spirituality and Religious Sentiments (140); BS, Between-Subject comparison; DB, Double-Blind; EQ, Experiences Questionnaire (141); FFMQ, Five Facets Mindfulness Questionnaire (142); FMI, Freiburg Mindfulness Inventory (143); IOS, Inclusion of Others in the Self scale (144); LCI-R, Life Changes Inventory (135); LOT-R, Life-Orientation Test-Revised (145); MEDEQ, Meditation Depth Questionnaire (146); NAS, Non-attachment Scale (147); NEO PI-R, Revised NEO Personality Inventory (148); PC, Placebo-Controlled; R, Randomised; TMS, Toronto Mindfulness Scale (149); WS, Within-subject comparison]*.

Psilocybin administration, following 1 month of preparatory training of spiritual practises, and spiritual support, led to a significant long-term increase in the Buddhist notion of non-attachment, describing an understanding of the impermanent nature of inner experiences, compared to baseline, assessed at the 6-month follow-up (60). This combined design also produced significantly higher enhancements in Spirituality, daily transcendental experience [as measured by the Daily Spiritual Experience scale; (151)] and positive attitudes about oneself, compared to those achieved by the control group. Importantly, the combination of high-dose psilocybin administration with high-intensity spiritual support nearly doubled long-term engagement with mindfulness meditation, spiritual awareness practise and journal writing, as reported at 6 months. Thus, psilocybin seems to accentuate the effects of mindfulness-training on trait mindfulness and enhance adherence to formal mindfulness meditation practises.

Trait mindfulness enhancements, following psychedelic administration have also been reported for a variety of substances, even in the absence of formal mindfulness-enhancing training (Summarised in Table 2). Post-acute enhancements in decentring, referring to the capacity to observe one's inner experiences in a detached manner, similarly to non-attachment, were demonstrated following ayahuasca administration (56, 57). Furthermore, enhanced acceptance towards present-moment thoughts and emotions has been observed post-acutely following the administration of ayahuasca and 5-MeO-DMT, which was maintained for up to 1 month (40, 52, 56–59). Individual studies also reported transient increases in self-compassion and present-moment awareness, as well as in the capacity to observe present-moment experience (40, 57, 58). Certain discrepancies and inconsistencies exist between these studies, even when the same psychedelic was administered. This points to the preliminary nature of these results, as most studies were uncontrolled and recruited quite modest sample sizes.

Taken together, these findings suggest that psychedelics could help novices to adhere to meditation training and could potentially accelerate this stage. Many novice meditators report finding meditation training particularly difficult (152, 153) and express uncertainty on whether they are “doing it right” (154), which could lead to them abandoning their practise. Although the link between trait mindfulness and mindfulness meditation remains largely inconclusive (155), some studies suggest that trait mindfulness predisposes for higher meditation depth (156), and so may help novice meditators remain engaged during practise. Furthermore, the acute experience of psychedelics may introduce people to altered states of consciousness which can also be achieved through deep meditation. In support of this, in a special psychedelic edition, the American Buddhist magazine *Tricycle: The Buddhist Review* included results from a poll titled “Psychedelics: Help or Hindrance?”, which showed that over 40% of readers' interest in Buddhism was sparked by psychedelics, and 71% of readers agreed with the statement “Psychedelics can provide a glimpse of the reality to which Buddhist practise points” (157).

Psychedelics could present as a useful adjunct to meditation for long-term meditators as well. Smigielski et al. demonstrated that psilocybin administration was able to enhance meditation depth [measured by the Meditation Depth Questionnaire (MEDEQ), (146)] post-acutely, and trait mindfulness [as measured by the Toronto Mindfulness Scale (TMS); (149)] long-term, even in their experienced meditator sample, who had over 5,000 h of meditation experience (61). Several long-term meditators report finding it difficult to advance their practise and “delve deeper” after years of experience. For those cases, psilocybin could be a useful adjunct to further their practise. As a case example, a long-term meditator engaging in “Ritual Meditation,” where mindfulness meditation was combined with truffles, reported a higher depth achieved during meditation and a sustained “inner peace and tranquillity” (Report 103668 from Erowid.org). In Douglas Osto's (158) online surveys of Western Buddhists, 49.3% supported that “Buddhism and psychoactive substance use are compatible,” and individual interviews revealed that a plethora of Buddhist meditators used psychedelics long-term, in conjunction with meditation (158).

These findings also demonstrate that trait changes in mindfulness capacities can be achieved pharmacologically, without any mindfulness training. The enhanced scores in trait mindfulness achieved in some studies after psychedelic administration (56, 57) were comparable and, in some cases, higher than those reported by a sample of long-term mindfulness meditators (98). A particularly striking result was that ayahuasca administration led to increases in the MINDSENS composite index (56, 57), which is particularly sensitive to the effects of meditation practise (52, 98). The enhancements demonstrated in these studies manifested rapidly, within 24 h post-administration, whilst enhancements in trait mindfulness mainly result from a long-term meditation practise, and can take longer to achieve in some individuals (159). Thus, individuals who experience difficulties maintaining a disciplined mindfulness meditation practise may still be able to reap some of the health benefits associated with it *via* the consumption of psychedelics, even from a single psychedelic administration. However, it's important to note that these enhancements in mindfulness were mostly transient, and so may need to be embedded into a theoretical or practical framework of mindfulness-training to be extended in time.

## Safety, Legal, and Ethical Considerations

Psychedelic-assisted therapy and mindfulness meditation have both been associated with various health, legal and ethical issues which must be critically considered in these respective fields. Psychedelics are considered safe substances, when administered in a supportive environment by clinicians, with a high potential for therapeutic benefits (50, 160). Although considerable stigma is associated with this class of drugs, very few studies have reported long-term adverse effects as a result of psychedelic administration, and in almost all cases where adverse reactions were reported, they were attributed to the unsupportive context in which they were administered (34, 41, 160). Furthermore, although some addictive potential has been demonstrated for classical and non-classical psychedelics on rat models (161), psychedelics do not lead to addiction in humans (162), and the rapid tolerance effects that follow psychedelic administration further limits their abuse potential (163, 164).

Mindfulness meditation has been generally deemed safe, but numerous reports exist of acute and long-term negative effects associated with the practise (74, 165–167). In most cases, underlying psychiatric conditions explained these adverse effects (168), but in other cases the negative experiences were thought to arise due to a negative ruminative pattern that had been reinforced *via* incorrectly taught and practised meditation (5, 167). Thorough instructions, a gradual immersion into formal practise and Neurofeedback-Assisted Mindfulness training have all been associated with higher meditation quality in meditation novices and experts (5, 169, 170) and could be applied to help minimise these adverse effects.

In recent studies which administered psychedelics within a mindfulness context, reviewed in the previous section (Three Recent Studies of a Combined Methodology) negative acute effects of psychedelics were minimised, and a deeper meditative state was achieved (60, 61, 171). Negative effects of meditation are thought to result from divided attention away from the meditation goal and rumination (167), especially in novices, and so enhancing meditation depth could minimise those effects. This preliminary evidence thus suggests that the combination of these two interventions might boost the safety profile of both and minimises adverse effects.

The legal status of psychedelic drugs, listed as “Class A” drugs, greatly limits scientific research into their therapeutic effects. Circumventing the legal status of psychedelics, listed among the most dangerous psychoactive substances, greatly hinders research efforts by delaying study onset, and may result in the premature cessation of experimental studies due to expenditure of funding for manufacturing costs, which can be considerable (172). Although policy change is imminent, using mindfulness training as an adjunct may help reduce the psychedelic dose required, as it can intensify the acute psychedelic experience (60, 61, 171), which is thought to mediate treatment effects (49). That way, manufacturing costs can be kept low and receiving approval from the ethics committee may prove easier.

Legal restrictions for the use of psychedelics also limit most scientific studies to the collection of data from naturalistic psychedelic ceremonies. This has ethical implications, as often the facilitators administering the drug have no medical or clinical background (40, 59), and so are unable to respond in a case of emergency and cannot exclude individuals with counterindications from the sessions.

## Conclusion and Future Directions

Preliminary findings from combined methodologies suggest that psychedelic-assisted therapy and mindfulness-based interventions have complementary effects on well-being and could potentially act as complementary adjuncts to enhance the salutogenic effects of either intervention. Mindfulness training intensified positively experienced drug-induced ego-dissolution, whilst psychedelics were able to enhance meditation depth and engagement with formal spiritual practises in expert and novice meditators, respectively. Furthermore, psychedelic administration within a naturalistic setting produced spontaneous enhancements in mindfulness capacities, which may also encourage or aid the practise of contemplative practises.

However, these studies suffer from methodological limitations and thus the effects may be overstated, especially as both fields of psychedelics and meditation are characterised by strong bias effects (11, 173). These findings require replication by studies with larger sample sizes and better-controlled methodology to conclusively demonstrate a synergistic relationship between mindfulness training and psychedelics. Future studies should aim to establish what salutogenic mechanisms underlie the therapeutic benefits of either interventions and propose a theoretical framework of how these might interact, when psychedelic administration is combined with mindfulness meditation. Additionally, a pilot study testing the potentially complementary effects of psilocybin and mindfulness meditation on depression could be particularly fruitful, as both interventions have displayed a plethora of anti-depressive mechanisms (51).

## Author Contributions

ME wrote the manuscript. TE helped conceive and critically revise the manuscript at all stages of production.

## Conflict of Interest

The authors declare that the research was conducted in the absence of any commercial or financial relationships that could be construed as a potential conflict of interest.

## Publisher's Note

All claims expressed in this article are solely those of the authors and do not necessarily represent those of their affiliated organizations, or those of the publisher, the editors and the reviewers. Any product that may be evaluated in this article, or claim that may be made by its manufacturer, is not guaranteed or endorsed by the publisher.
